# Scutellarin Exerts Hypoglycemic and Renal Protective Effects in db/db Mice via the Nrf2/HO-1 Signaling Pathway

**DOI:** 10.1155/2019/1354345

**Published:** 2019-02-10

**Authors:** Yange Liu, Juan Wang, Xinrui Zhang, Li Wang, Tian Hao, Yanli Cheng, Di Wang

**Affiliations:** ^1^School of Life Sciences, Jilin University, Changchun 130012, China; ^2^Academy of Science, Liaoning University, Shenyang 110036, China; ^3^Department of Nephrology, The First Hospital of Jilin University, 71 Xinmin Street, Changchun, 130021 Jilin Province, China

## Abstract

This study investigated the hypoglycemic and renal protective effects of scutellarin (SCU) in db/db mice and elucidated the underlying mechanisms. The oral administration of metformin hydrochloride (Met) at 120 mg/kg and SCU at 25, 50, and 100 mg/kg over an eight-week period had hypoglycemic effects, demonstrated by decreases in body weight, blood glucose, food and water intake, and glycated hemoglobin activity and by augmented insulin levels and pyruvate kinase activity in the serum of db/db mice. SCU alleviated dyslipidemia by decreasing the levels of triglycerides and total cholesterol and enhancing the levels of high-density lipoprotein cholesterol in the serum of db/db mice. SCU reversed the overexpression of mRNA of renal damage markers (receptor for advanced glycation end products, neutrophil gelatinase-associated lipocalin, and kidney injury molecule 1), macrophage marker CD11b, and T cell marker CD3 in kidney of db/db mice. Pathological examination confirmed that SCU improved the organ structures of hyperglycemia-damaged livers, kidneys, and pancreas islets. Antibody array assay and enzyme-linked immunosorbent assay were combined to screen and analyze the regulatory effects of SCU on inflammatory factors and oxidative enzymes. SCU exerted anti-inflammatory effects by inhibiting the levels of proinflammatory cytokines (glycogen synthase kinase, intercellular adhesion molecule 2, and interleukin 1*β* and 2) and promoting anti-inflammatory cytokines (interleukin 4). SCU decreased the reactive oxygen species and malondialdehyde concentrations and increased the activity levels of antioxidative enzymes (superoxide dismutase, glutathione peroxidase, and catalase) in serum and kidneys. Furthermore, SCU upregulated the expression of nuclear factor erythroid 2-related factor 2 (Nrf2), which in turn improved heme oxygenase 1 (HO-1), superoxide dismutase 1 and 2, and catalase expression levels in kidneys. The study showed that SCU has at least partial hypoglycemic and renal protective effects in db/db mice, and the mechanism is the modulation of the Nrf2/HO-1 signaling pathway.

## 1. Introduction

As a chronic metabolic disorder, diabetes mellitus (DM) is a major threat worldwide [1]. The impaired homeostasis of the carbohydrate and lipid metabolism is a universal feature of DM, which ultimately results in impaired glucose tolerance, insulin resistance, and hyperglycemia [2]. Type 2 diabetes mellitus is the most common type, accounting for 90% of the cases; the remaining 10% are primarily gestational diabetes and type 1 diabetes mellitus [3]. Prolonged hyperglycemia leads to a series of complications for type 2 patients. Diabetic nephropathy (DN), which is a leading cause of end-stage renal disease, is the most common diabetic microvascular complication, and it is associated with high mortality and morbidity [4].

As DM progresses, the amount of inflammation is closely related to the exorbitant cytokine concentrations secreted by the activated immune cells [5]. In a vicious cycle, the inflammatory molecules recruit lots of mononuclear cells to the injury site, which further exacerbates DM [6] and leads to tubulointerstitial fibrosis and renal hypertrophy [7]. Under hyperglycemic conditions, the abnormal accumulation of reactive oxygen species (ROS) leads to cellular damage by disrupting DNA and hampering normal mitochondrial function, which triggers the occurrence of oxidative stress [8]. The overproduction of ROS enhances inflammatory responses in diabetic patients [9]. Nuclear factor erythroid 2-related factor 2 (Nrf2) is a master regulator of cellular antioxidant activity that activates the expression of various genes involved in antioxidative defenses [10]. Sodium butyrate, a known activator of Nrf2, ameliorates diabetes-induced renal oxidative damage, pathological changes, and dysfunction [11], which suggests that Nrf2 has a key role in the pathogenesis of DN. The overexpressions of catalase (CAT), heme oxygenase 1 (HO-1), and superoxide dismutase (SOD) have been found to protect *β*-cells from deleterious combinations of cytokines, indicating the important role of oxidative stress in inflammation-associated demise under DM, and even DN [12].

Metformin (Met), the commonly used drug for DM, could promote pancreatic *β*-cell functions and decrease hepatic glucose production and intestinal glucose absorption [13]. Multiple natural compounds with various biological activities have become a treasure trove for researchers developing new drugs. In our group, we have successfully confirmed the hypoglycemic and renal protective effects of *Inonotus obliquus* polysaccharides and *Paecilomyces hepiali* mycelium through the modulation of oxidative stress and inflammatory factors [14, 15].

Scutellarin (SCU, 4,5,6-trihydroxyflavone-7-glucuronide), a flavone mainly obtained from *Erigeron breviscapus (vant.) Hand. Mazz.*, possesses pharmacological properties such as anti-inflammation [16], antioxidant effects [17], and the inhibition of adipogenesis [18]. SCU exerts antioxidant effects via Nrf2 nuclear translocation and has been found to enhance the expression levels of heme oxygenase 1 (HO-1) [19]. SCU-loaded Chit-DC-VB12 nanoparticles have been found to downregulate the central retinal artery resistivity index and angiogenesis-related proteins' expressions of retinas in type 2 diabetic rats [20]. Additionally, SCU significantly inhibits hyperglycemia-induced apoptotic cells and morphologic impairments in testes of rats [21]. Although SCU has been used to treat some type 2 diabetes mellitus-induced complications, the hypoglycemic and renal protective effects of SCU in DM have not been systematically studied.

BKS.Cg-Dock7^m^ +/+ Lepr^db^/JNju mice (db/db mice) carry a gene mutation in the leptin receptor and have been widely used for studies of type 2 diabetes mellitus [22, 23]. At 12-14 weeks of age, the db/db mice had glomerular hypertrophy and mesangial cell proliferation, which can be used as an animal model of diabetic nephropathy [24]. This study investigated the hypoglycemic and renal protective effects of SCU in db/db mice, which may be related to its modulation of the Nrf2/HO-1 signaling pathway.

## 2. Methods and Materials

### 2.1. Animal Experiment Design

**Figure 1:**
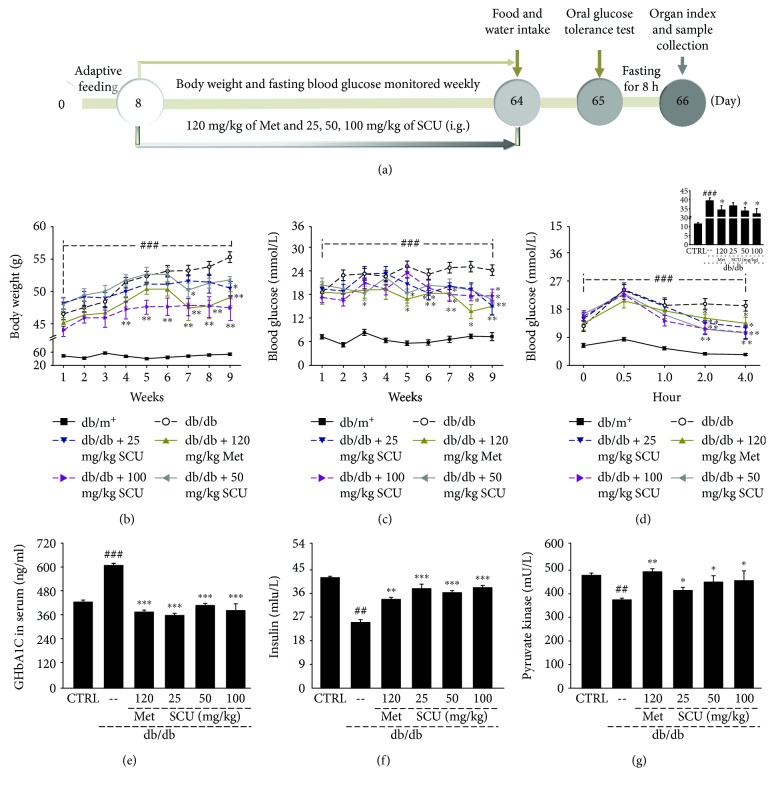
(a) Schematic of the animal experimental protocol and drug administration. Eight weeks of SCU and Met treatment regulated (b) body weight, (c) blood glucose, (d) glucose tolerance, and the levels of (e) glycated hemoglobin, (f) insulin, and (g) pyruvate kinase in serum of db/db mice. Results are represented as means ± SEM (*n* = 8). ^##^ *P* < 0.01 and ^###^ *P* < 0.001 in a comparison with the db/m^+^ mice, ^∗^ *P* < 0.05, ^∗∗^ *P* < 0.01, and ^∗∗∗^ *P* < 0.001 in a comparison with the vehicle-treated db/db mice. SCU: scutellarin; Met: metformin.

The experimental protocols were approved by the Institution Animal Ethics Committee of Jilin University (Reference No. 20160302). The process of model development and drug treatment processes were similar to those used in previous studies with some modifications [23, 25, 26]. Forty male BKS.Cg-Dock7^m^ +/+ Lepr^db^/JNju mice (db/db mice, 7 weeks) and eight nondiabetic C57BLKS/J-LepR^db/+^ mice (db/m^+^ mice, 7 weeks) were purchased from the Model Animal Research Center of Nanjing University (Nanjing, China). All of the mice were housed in an environmentally controlled room (temperatures maintained at 23 ± 1°C, relative humidity of 55% ± 5%, 12 h dark/12 h light cycle). After 1 week of adaptive feeding, the db/db mice were randomly divided into five groups (*n* = 8/group) and orally treated with 10 mL/kg of normal saline (model group), metformin hydrochloride at 120 mg/kg (positive control group), and SCU at doses of 25, 50, and 100 mg/kg (SCU-treated groups) for 8 consecutive weeks. The db/m^+^ mice (control group) were orally treated with 10 mL/kg of normal saline for eight consecutive weeks. Body weight and fasting blood glucose were monitored weekly during the experiments. The details of the experimental protocol and drug administration are shown in Figure 1(a). Animals were individually housed in metabolic cages for 24 h, and the volumes of food and water intake were measured.

### 2.2. Oral Glucose Tolerance Test

After the 8-week administration period, all of the mice were fasted for 12 h (20:00 to 8:00) and their blood glucose was measured in blood samples taken from the tail vein. Then, the mice were orally treated with 2.0 g/kg of glucose, and their blood glucose levels were measured at 0.5 h, 1.0 h, 2.0 h, and 4.0 h. The glucose area under the curve at the baseline was calculated using the following formula:

(1) $$\begin{array}{c} \text{AUC}=\left(0\text{ h blood glucose}+0.5\text{ h blood glucose}\right)\times 0.25+\left(0.5\text{ h blood glucose}+1.0\text{ h blood glucose}\right)\times 0.25+\left(1.0\text{ h blood glucose}+2.0\text{ h blood glucose}\right)\times 0.5. \end{array}$$

### 2.3. Sample Collection and Organ Index Test

After the oral glucose tolerance test, all mice were fasted (except for water) for 8 h and blood samples were collected from the caudal vein. At the end of the experiment, all of the mice were sacrificed, and their organs including heart, liver, pancreas, and kidney were harvested, weighed, and partially preserved at −80°C. The organ index was calculated using the following formula:

(2) $$\begin{array}{c} \text{Organ index }\%=\frac{\text{mean organ weight}}{\text{mean body weight}}. \end{array}$$

### 2.4. Histology Assay

The pancreas, liver, and kidney tissues were fixed in 4% phosphate-buffered formaldehyde, dehydrated in a gradient of ethanol, and then embedded in paraffin. 5 *μ*m sections were consecutively cut, deparaffinized in xylene, rehydrated in graded concentrations of ethanol, and stained by hematoxylin and eosin (H&E) for histological evaluation. All stained sections were visualized with a light microscope at ×400 magnification (IX73 inverted microscope, Olympus, Japan).

### 2.5. Antibody Array Assay

The L-series Mouse Antibody Array Kit, purchased from RayBiotech Inc. (AAM-BLG-1-2, USA), was used to detect the 308 cytokines in the kidney tissues collected from all of the groups. Each kidney sample's total protein was extracted with ice-cold Cell & Tissue Protein Extraction Reagent (KC-415, KangChen, China), which contains inhibitors for protein degradation (5 *μ*L PMSF, 5 *μ*L protease inhibitor cocktail, and 5 *μ*L phosphatase cocktail added to 1 mL protein extraction reagent). The protein concentration of each sample was measured using a BCA protein assay kit (KC-430, KangChen, China). Protein array membranes were blocked for 30 min in a blocking buffer, then incubated with samples at 4°C overnight. After washing, the membranes were incubated with diluted biotin-conjugated antibodies for 2 h at room temperature and then reacted with streptavidin-conjugated fluorescein at room temperature. Membranes were then scanned (GenePix 4300A, Axon, US).

### 2.6. Biochemical Index Measurement

The levels of triglyceride (TG), total cholesterol (TCHO), high-density lipoprotein cholesterol (HDL-C), glycogen synthase kinase (GSK), interleukin- (IL-) 1*β*, IL-2, IL-4, IL-6, IL-8, intercellular adhesion molecule- (ICAM-) 1, ICAM-2, monocyte chemotactic protein-5 (MCP-5), matrix metalloproteinase-9 (MMP-9), tumor necrosis factor-*α* (TNF-*α*), transforming growth factor-*β*1/2 (TGF-*β*1/2), interferon-*β* (IFN-*β*), CAT, glutathione peroxidase (GSH-Px), SOD, ROS, and malondialdehyde (MDA) in the serum and/or kidney were measured using enzyme-linked immunosorbent assay kits (Shanghai Yuanye Bio-Technology Co. Ltd., China) according to the manufacturer's instructions.

### 2.7. Reverse Transcription-Polymerase Chain Reaction (RT-PCR)

RT-PCR was performed according to a method described previously with some modifications [27]. Briefly, the RNA was isolated from the kidney using TRIzol (Invitrogen, USA) and then synthesized by QuantScript RT Kit (Tiangen Biotech (Beijing) Co. Ltd., China). GAPDH primers were used as an internal control. The conditions of PCR amplification was shown as follows: denaturation at 95°C for 5 min, followed by 36 cycles at 95°C for 45 s, 57°C for 45 s, and 72°C for 45 s. The primer sequences are listed in Table 1s.

### 2.8. Western Blot Analysis

Partial kidney tissues were thoroughly homogenized in a lysis buffer (0.97% protease inhibitor cocktail, 0.94% 50 mM phenylmethylsulfonyl fluoride, and 97.09% 1x RIPA) on ice. The protein concentrations of the homogenates were measured using the BCA Protein Assay Kit (Merck Millipore, USA); 50 *μ*g of protein was electrophoresed on 12% SDS-PAGE, transferred onto polyvinylidene difluoride (PVDF) membrane (Merck Millipore, USA), and blocked in 5% bovine serum albumin (BSA) in Tris-buffered saline. Then, the bands were incubated overnight at 4°C in a corresponding primary antibody solution containing Nrf2 (ab137550), HO-1 (ab13248), SOD1 (ab13498), SOD2 (ab13533), and CAT (ab16731) (1 : 2000; Abcam, UK) or glyceraldehyde 3-phosphate dehydrogenase (GAPDH, ABS16, 1 : 2000, Merck Millipore, USA) and then incubated with horseradish peroxidase-conjugated goat anti-rabbit secondary antibodies (bs-0295G, 1 : 2000, Beijing Biosynthesis Biotechnology Co. Ltd., China) for 4 hours at 4°C. Specific signals were visualized with ECL detection on a gel imaging system (UVP, California, USA). The average optical density of the bands was quantified using ImageJ (National Institutes of Health, Bethesda, USA).

### 2.9. Statistical Analysis

All the experimental data are expressed as mean ± SEM. A one-way ANOVA followed by post hoc multiple comparisons (Dunn's test) was used to evaluate significant differences between groups using SPSS 16.0 software (IBM Corporation, Armonk, USA), and *P* < 0.05 was interpreted as statistically significant.

## 3. Results

### 3.1. Hypoglycemic Effects of SCU in db/db Mice

Organ index changes can partially reflect physical conditions [28]. Compared with db/m^+^ mice, significant changes in the heart, spleen, and kidney indexes were noted in the 16-week-old db/db mice (*P* < 0.001; Table 1), but there were no significant changes in the liver index (Table 1). The only index enhanced in the Met and SCU groups was the heart index (*P* < 0.05; Table 1).

After the 8-week administration period, the high levels of food and water intake observed in the db/db mice were all strongly reversed in the Met and SCU groups at all of the tested doses (*P* < 0.001; Table 1).

Increased body weight and blood glucose were noted following the onset of diabetes [29]. 100 mg/kg SCU significantly lowered the body weight of db/db mice from the third week (*P* < 0.01; Figure 1(b)). Both Met and SCU suppressed the blood glucose of db/db mice after the 8-week administration period (*P* < 0.05; Figure 1(c)). Glucose tolerance is a body's capacity to mediate blood glucose concentration [30]. After oral doses of glucose, the blood glucose levels and glucose area under the curve (AUC) after 2 hours were significantly lower in the SCU- and Met-treated db/db mice than in the nontreated group (*P* < 0.05; Figure 1(d)), suggesting that these substances ameliorated glucose intolerance.

The concentrations of GHbA1c, insulin, and pyruvate kinase are correlated with glucose levels and therefore are viewed as dependable indexes for the diagnosis of diabetes [31]. Compared with db/m^+^ mice, enhanced levels of GHbA1c (*P* < 0.001, Figure 1(e)) and decreased insulin levels and pyruvate kinase activity (*P* < 0.01, Figures 1(f) and 1(g)) were observed in db/db mice; these levels were all strongly normalized by SCU and Met (*P* < 0.05, Figures 1(e)–1(g)).

### 3.2. Effects of SCU on Lipid Profiles in db/db Mice

**Figure 2:**
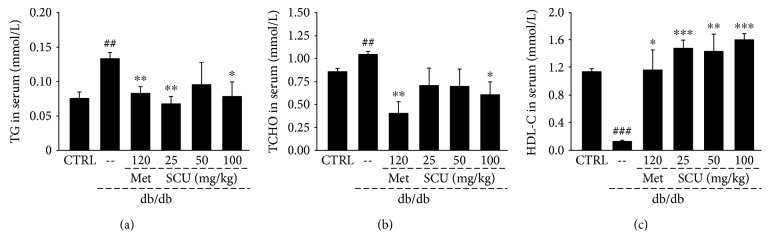
Eight weeks of SCU and Met treatment regulated the levels of (a) triglyceride, (b) total cholesterol, and (c) high-density lipoprotein cholesterol in serum of db/db mice. Results are represented as means ± SEM (*n* = 8). ^##^ *P* < 0.01 and ^###^ *P* < 0.001 in a comparison with the db/m^+^ mice, ^∗^ *P* < 0.05, ^∗∗^ *P* < 0.01, and ^∗∗∗^ *P* < 0.001 in a comparison with the vehicle-treated db/db mice. SCU: scutellarin; Met: metformin.

Dyslipidemia is frequently present in diabetic patients, particularly due to the poor control of blood glucose [32]. Compared with db/m^+^ mice, db/db mice had abnormally high levels of TG (*P* < 0.01, Figure 2(a)) and TCHO (*P* < 0.01, Figure 2(b)) and low levels of HDL-C (*P* < 0.001, Figure 2(c)) in serum. As in the Met group, the SCU group showed hypolipidaemic effects in the db/db mice such as the suppression of TG and TCHO levels and the enhancement of HDL-C levels (*P* < 0.05, Figure 2).

### 3.3. The Protection of SCU on the Liver, Kidney, and Pancreas in db/db Mice

**Figure 3:**
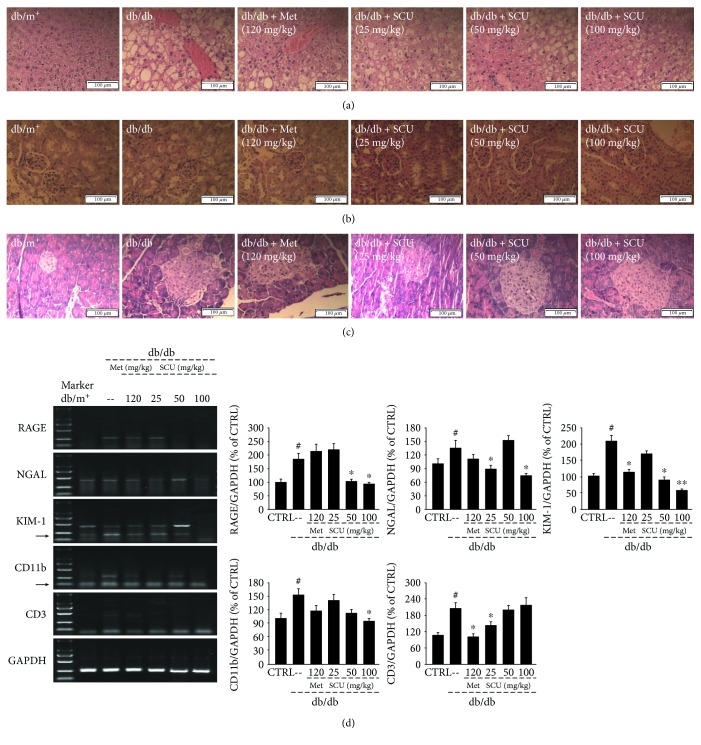
Histopathological analysis in the (a) liver, (b) kidney, and (c) pancreas by H&E staining (scale bar: 100 *μ*m; magnification: 400x). (d) mRNA expression of the renal damage markers RAGE, NGAL, and KIM-1, macrophage marker CD11b, and T cell marker CD3 in the kidneys of db/db mice measured using RT-PCR. Marker size from top to bottom: 2000 bp, 1000 bp, 750 bp, 500 bp, 250 bp, and 100 bp. H&E: hematoxylin and eosin; RAGE: receptor for advanced glycation end products; NGAL: neutrophil gelatinase-associated lipocalin; KIM-1: kidney injury molecule 1; RT-PCR: reverse transcription-polymerase chain reaction. ^#^ *P* < 0.05 in comparison with db/m^+^ mice, ^∗^ *P* < 0.05 and ^∗∗^ *P* < 0.01 in comparison with vehicle-treated db/db mice.

Lipid accumulation in the vesicles of hepatocytes and the fatty degeneration of hepatocytes was detected in the liver of db/db mice, but this was attenuated in the SCU and Met groups, as suggested by the extreme reduction in the formation of fat vacuoles (Figure 3(a)). A glomerular hypertrophy, that is, a thickening of the basement membrane and a narrowing of the capsular space of the kidney, and irregularly shaped pancreatic islets were noted in db/db mice compared to db/m^+^ mice (Figures 3(b) and 3(c)). All of these pathological changes were strongly relieved by SCU and Met (Figures 3(b) and 3(c)).

Hyperglycemia induces serious diabetic microvascular complications such as nephropathy. Neutrophil gelatinase-associated lipocalin (NGAL), kidney injury molecule 1 (KIM-1), and the receptor for advanced glycation end products (RAGE) are biomarkers of renal injury that can predict early diabetic nephropathy [33, 34]. The elevated NGAL, KIM-1, and RAGE mRNA levels in db/db mice were reduced in the SCU-treated group, especially at 100 mg/kg (*P* < 0.05, Figure 3(d)).

Macrophages and T cells play an important role in inflammation [35]. The gene expressions of CD11b, a characteristic macrophage marker, and CD3, a characteristic T cell marker, were significantly upregulated in db/db mice compared with db/m^+^ mice (*P* < 0.05, Figure 3(d)). Treatment with SCU inhibited the decrease in CD11b and CD3 gene expression in db/db mice compared to the vehicle-treated db/db mice (*P* < 0.05, Figure 3(d)).

### 3.4. Renal Protection of SCU via Regulation of Inflammatory Factors in db/db Mice

**Figure 4:**
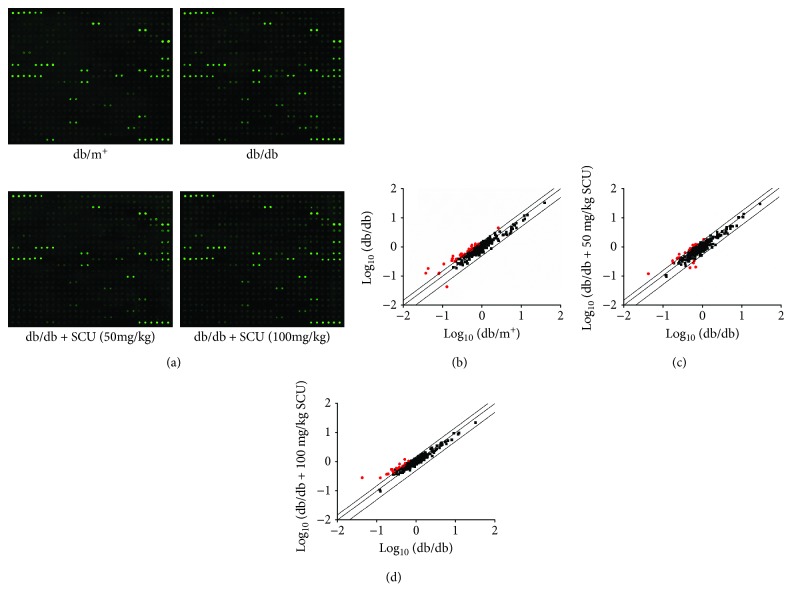
The effects of SCU on the 308 cytokines in kidney of db/db mice were detected by the Mouse Cytokine Array Kit (*n* = 3). (a) The fluorescent graphical representation of cytokine expressions. (b, c and d) Scatter diagram of the 308 cytokines. The relative density is the ratio of the absolute value and the reference spot value. The red dots indicate the factors with a change of >50% (db/db mice vs. db/m^+^ mice and SCU-treated db/db mice vs. vehicle-treated-db/db mice).

Fluorescent images performed using a comprehensive biotin label-based cytokine tip assay suggested that 28 of the 308 target cytokines related to inflammation were strongly enhanced, and 1 was decreased over 50% in the vehicle-treated db/db mice compared with the db/m^+^ mice (Figure 4 and Table 2s). Compared with vehicle-treated db/db mice, 50 mg/kg doses of SCU increased by over 50% the levels of 15 types of cytokines and reduced the levels of 4 types of cytokines in db/db mice. Doses of 100 mg/kg of SCU strongly regulated 24 types of cytokines (Figure 4 and Table 2s).

Based on the results of the cytokine array assay, 13 pro- and anti-inflammation cytokines were further analyzed using the ELISA method. Hyper-levels of GSK (*P* < 0.01) and ICAM-2 (*P* < 0.05) in serum and of IL-1*β* (*P* < 0.05) and IL-2 (*P* < 0.05) in kidneys and hypo-levels of IL-4 in kidneys (*P* < 0.05) were found in db/db mice compared with db/m^+^ mice (Table 2). Compared with vehicle-treated db/db mice, db/db mice after eight weeks of SCU administration had a 12% reduction in the serum levels of GSK (*P* < 0.05), 8.1% (*P* < 0.05) and 13.6% (*P* < 0.05) reductions in the levels of IL-1*β* and IL-2 in kidneys, and a 33.5% (*P* < 0.01) increase in the renal levels of IL-4 (Table 2). Additionally, SCU significantly decreased the serum levels of ICAM-2 by up to 26.7%, but failed to influence its renal levels (Tables 2 and 2s). SCU and Met treatment failed to influence the levels of MMP-9, TNF-*α*, IL-6, IL-8, ICAM-1, MCP-5, TGF-*β*1/2, and IFN-*β* in the serum and/or kidneys of db/db mice (Table 3s).

### 3.5. SCU Displayed an Antioxidative Effect by Regulating Nrf2/HO-1 Signaling

Compared with db/m^+^ mice, the extremely low levels of CAT, GSH-Px, and SOD in serum and kidney of db/db mice (*P* < 0.05; Table 3) were markedly prevented by an 8-week administration of SCU, especially at a dose of 100 mg/kg (*P* < 0.05; Table 3). Compared with db/m^+^ mice, db/db mice exhibited a 10.8% enhancement of renal ROS levels (*P* < 0.05; Table 3); however, no significant changes in levels of MDA were observed. The administration of SCU over an 8-week period resulted in a >19.6% reduction in ROS levels in the kidneys (*P* < 0.05) and a >11.1% reduction in MDA levels in the serum (*P* < 0.01) and kidneys (*P* < 0.05) of db/db mice (Table 3).

**Figure 5:**
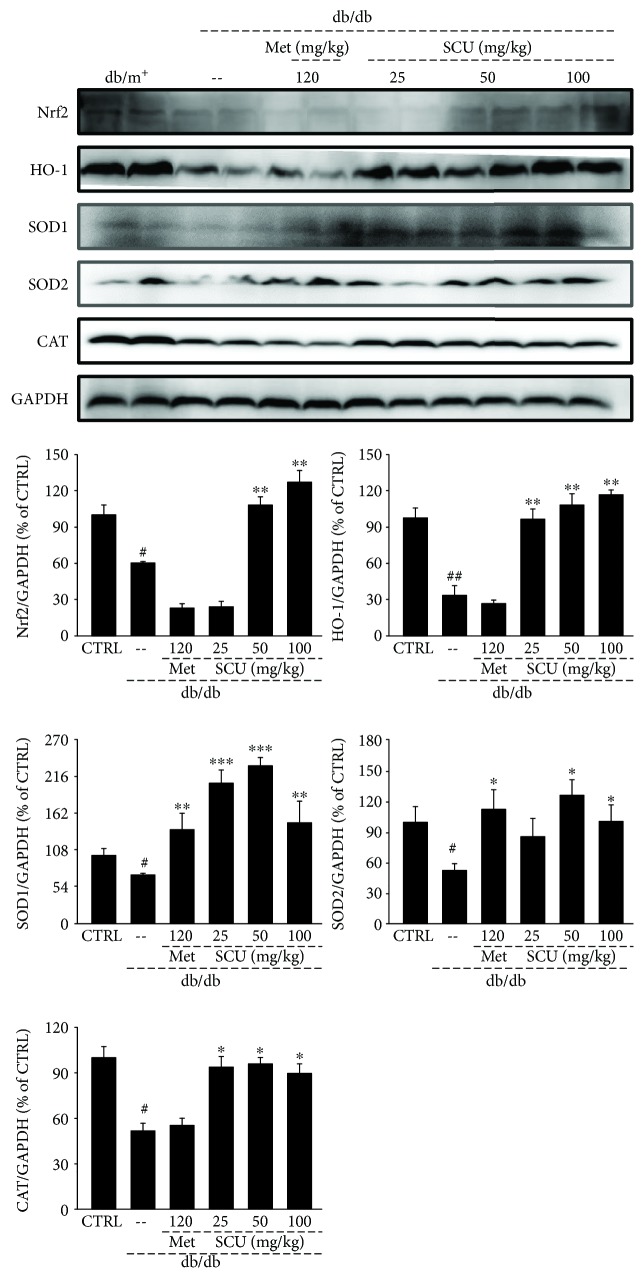
Four weeks of SCU treatment increased the expression levels of Nrf2, HO-1, SOD1, SOD2, and CAT in the kidney of db/db mice. The data on quantified protein expressions were normalized by related glyceraldehyde-3-phosphate dehydrogenase. The results are represented as means ± SEM (*n* = 4). ^#^ *P* < 0.05 and ^##^ *P* < 0.01 in a comparison with the db/m^+^ mice, ^∗^ *P* < 0.05, ^∗∗^ *P* < 0.01, and ^∗∗∗^ *P* < 0.001 in a comparison with the vehicle-treated db/db mice. SCU: scutellarin; Met: metformin.

Concurrent with the 39.7% decrease in Nrf2 expression levels (*P* < 0.05), the expression levels of HO-1 (*P* < 0.01), SOD1 (*P* < 0.05), SOD2 (*P* < 0.05), and CAT (*P* < 0.05) were significantly depressed in the kidneys of db/db mice by up to 66%, 29.2%, 47%, and 48.5%, respectively, compared with the db/m^+^ mice (Figure 5). The SCU administration enhanced the expression levels of Nrf2 (*P* < 0.01) and further led to the increased activation of HO-1 (*P* < 0.01), SOD1 (*P* < 0.01), SOD2 (*P* < 0.05), and CAT (*P* < 0.05) in db/db mice (Figure 5). Met only enhanced the expression levels of SOD1 (*P* < 0.01) and SOD2 (*P* < 0.05) (Figure 5).

## 4. Discussion

In db/db mice, SCU increases hypoglycemic activities such as alleviating food and water intake, effectively reducing body weight and fasting blood glucose, raising glucose tolerance, and inhibiting the level of GHbA1c. GHbA1c is the product of the un-reversible combination of hemoglobin and blood glucose in red blood cells, which is positively correlated with blood glucose concentration [36]. Insulin, produced by *β*-cells, regulates blood glucose levels by converting glucose into glycogen [37]. Dysfunction of the pancreatic islets and low secretion of insulin are major indicators of DM. Insulin promotes the synthesis of PK, one of the main rate-limiting enzymes in glycolysis, which can catalyze enolphosphopyruvate and ADP to ATP and pyruvate [38]. This study found that SCU effectively reduces the irregular shape of pancreatic islets and enhances the levels of insulin and PK activity, further confirming its capacity to mitigate the hyperglycemia burden in db/db mice. Hyperglycemia promotes the synthesis of TG and TCHO and reduces the HDL-C level in the liver, which results in lipid metabolism disorders in diabetic patients [39]. The excess free fatty acids in the blood produced by the irregular lipid metabolism accumulate in the liver [40]. Encouragingly, our results confirmed the beneficial effects of SCU in attenuating dyslipidemia in db/db mice.

Hyperglycemia causes renal hemodynamic changes and metabolic abnormalities that lead to renal injury through the upregulation of the production of proinflammatory cytokines [41]. The renal protection of SCU has been further confirmed by its suppression on the mRNA levels of NGAL, KIM-1, and RAGE in the kidney. NGAL and KIM-1 reflect the tubular damage associated with the collateral tubulointerstitial inflammation in glomerulonephritis/vasculitis [33]. Advanced glycation-end products (AGEs) form a regulatory network by binding to and activating its specific receptor for AGEs (RAGE), which boost intracellular signaling transductions, inducing renal cell proliferation and eventually accelerating the pathological progression of diabetic renal fibrosis [34]. This inflammatory activation leads to impaired insulin secretion and function [42], which further exacerbate diabetes. Adipose tissue inflammation and islet inflammation are associated with increased macrophage numbers [43, 44], which trigger immune response [45]. G-CSF stimulates the maturation of granular and mononuclear macrophages and is closely related to inflammation response. ICAM-2 influences NK cell-mediated clearance, adhesive interactions for antigen-specific immune response, and lymphocyte recirculation [46]. IL-1*β* helps to increase the secretion of G-CSF [47] and ICAM-1 [48], which is responsible for *β*-cell damage and death in islets, and further aggravates DM symptoms in rodents and humans [5, 49]. On the other hand, TGF-*β* suppresses the synthesis of proinflammatory molecules, such as IL-2, alleviates renal fibrosis [50], and prevents the IL-1*β*-dependent proliferation of activated T cells [51]. The activation of T cell results in secretion of proinflammatory effector interleukins such as IL-1*β* and IL-2 [52]. This imbalance of IL-1*β* leads to pancreatic islet inflammation [53], aggravating diabetes mellitus. Similarly, lipopolysaccharide stimulates the secretion of inflammatory factors, such as TNF-*α* and IL-1*β*, in the macrophage [54], which causes the development of inflammatory responses. Additionally, the significant elevation in IL-2 level was also noted in patients with nephropathy [55]. IL-4 could inhibit IL-2-induced activation of NK and show anti-inflammatory roles based on its protective effects in diabetes [56]. SCU affected the function of T cell and macrophage to ameliorate hyperglycemia-induced inflammation in the kidney of db/db mice.

Hyperglycemia and fatty acid oxidation-mediated oxidative stress are the foundation for the development of DM, which occurs when there is an overaccumulation of ROS due to low levels of antioxidant genes [57]. Excessive production of ROS promotes progressive metabolic and mitochondrial dysfunction leading to oxidative stress, which can reduce insulin secretion from *β*-cells [58]. Furthermore, the overexpression of ROS has been found to increase with proinflammatory cytokines, which act as signaling molecules and mediators for inflammatory responses [59]. Nrf2 regulates redox homeostasis, plays a critical role in preventing oxidative stress, and exhibits the potential to be a prospective target for diabetes. Nrf2 helps to activate specific genes including HO-1 and SOD [57]. Of these genes, SOD, an important enzymatic cellular antioxidant, contains three main variants located in specific cellular sites including the cytosol (Cu–Zn-SOD, SOD1), mitochondria (Mn-SOD, SOD2), and extracellular space [60]. SOD converts O_2_^−^ into the less reactive H_2_O_2_ radical. Although H_2_O_2_ is known to be harmful, CAT and peroxidase immediately break H_2_O_2_ down into H_2_O. HO-1, a widespread antioxidative enzyme, catalyzes free heme into carbon monoxide, biliverdin, and ferrous iron and is helpful in suppressing inflammation [61]. The antioxidative enzymes are responsible for scavenging free radicals, maintaining redox homeostasis, and decreasing increases in ROS, which depresses inflammatory responses and thus DM [62]. The antioxidative effects of SCU were confirmed by cytokine detection and protein analysis, which controlled the hyperglycemia and inflammatory response in db/db mice.

## 5. Conclusion

Compared with previous research, this study first systematically investigates the hypoglycemic and renal protective effects of SCU in db/db mice and confirms that SCU have effective actions against DM by controlling blood glucose concentration and insulin secretion and mitigating abnormal lipid accumulation and renal inflammation at least partially via modulation of the Nrf2/HO-1 signaling pathway.

## Figures and Tables

**Table 1:** The effect of SCU and Met on organ indexes, food intake, and water intake in db/db mice.

		db/m^+^	db/db	db/db + Met (mg/kg)	db/db + SCU (mg/kg)		
				120	25	50	100
Organ index %	Liver	4.5 ± 0.8	5.5 ± 0.2	5.1 ± 0.3	4.5 ± 0.6	5.4 ± 0.3	5.3 ± 0.3
	Heart	0.58 ± 0.04	0.28 ± 0.009^###^	0.31 ± 0.01^∗^	0.31 ± 0.02	0.32 ± 0.02^∗^	0.34 ± 0.02^∗∗^
	Spleen	0.31 ± 0.008	0.15 ± 0.02^###^	0.15 ± 0.03	0.14 ± 0.01	0.14 ± 0.007	0.15 ± 0.01
	Kidney	1.42 ± 0.04	0.75 ± 0.02^###^	0.84 ± 0.06	0.76 ± 0.01	0.74 ± 0.02	0.78 ± 0.04

Food intake (g/100 g)		8.7 ± 1.3	22.1 ± 0.8^###^	13.4 ± 0.6^∗∗∗^	10.9 ± 1.1^∗∗∗^	12.8 ± 1.4^∗∗∗^	14.0 ± 1.8^∗∗∗^
Water intake (g/100 g)		27.9 ± 1.6	65.4 ± 4.1^###^	37.6 ± 3.3^∗∗∗^	32.7 ± 2.7^∗∗∗^	36.0 ± 2.5^∗∗∗^	34.0 ± 5.0^∗∗∗^

^###^ *P* < 0.001 versus db/m^+^ mice, ^∗^ *P* < 0.05, ^∗∗^ *P* < 0.01, and ^∗∗∗^ *P* < 0.001 versus vehicle-treated db/db mice.

**Table 2:** The regulatory effects of SCU and Met on the levels of inflammatory cytokines.

		db/m^+^	db/db	db/db + Met (mg/kg)	db/db + SCU (mg/kg)		
				120	25	50	100
Serum	GSK (pmol/L)	490.9 ± 13.6	570.5 ± 17.8^##^	446.4 ± 10.7^∗∗^	510.4 ± 15.6	496.1 ± 23.1^∗^	502.1 ± 12.8^∗^
	ICAM-2 (nmol/mL)	29.4 ± 0.6	35.2 ± 1.4^#^	26.6 ± 1.6^∗^	19.3 ± 1.6^∗∗∗^	23.3 ± 0.9^∗∗^	25.8 ± 1.2^∗^

Kidney	IL-1*β* (pg/mgprot)	34.1 ± 2.1	37.2 ± 2^#^	34.5 ± 1.6	31.8 ± 2.4^∗^	34.2 ± 2.1^∗^	35.1 ± 0.5
	IL-2 (pg/mgprot)	74.4 ± 3	84.8 ± 4.4^#^	85.8 ± 2.6	72.8 ± 5^∗^	73.3 ± 1.1^∗^	65.3 ± 2.3^∗∗^
	IL-4 (pg/mgprot)	37.9 ± 1.6	32.5 ± 2.1^#^	40.5 ± 0.6^∗^	43.4 ± 4.2^∗∗^	33.8 ± 1.3	37.7 ± 1.2

^#^ *P* < 0.05 and ^##^ *P* < 0.01 versus db/m^+^ mice, ^∗^ *P* < 0.05, ^∗∗^ *P* < 0.01, and ^∗∗∗^ *P* < 0.001 versus vehicle-treated db/db mice.

**Table 3:** The regulatory effects of SCU and Met on the levels of oxidative cytokines.

		db/m^+^	db/db	db/db + Met (mg/kg)	db/db + SCU (mg/kg)		
				120	25	50	100
Serum	CAT (U/mL)	71.8 ± 0.5	52.5 ± 1.5^##^	54.2 ± 1.4	56.2 ± 2.2	54.4 ± 0.7	59.3 ± 1.3^∗^
	GSH-Px (U/mL)	293.7 ± 10.2	249.3 ± 5.1^#^	265.5 ± 6.8	267.5 ± 10.3	267.9 ± 3.9	310.5 ± 13.8^∗∗^
	SOD (U/mL)	250.8 ± 12	137.5 ± 21.5^###^	206.8 ± 6.9^∗∗^	196.2 ± 16.4^∗^	200.4 ± 12.6^∗^	206.9 ± 15.6^∗∗^
	MDA (nmol/mL)	21.2 ± 0.4	20.1 ± 0.6	17.2 ± 0.6^∗^	16.1 ± 0.5^∗∗^	15.3 ± 0.5^∗∗^	16.2 ± 0.5^∗∗^

Kidney	CAT (U/mgprot)	22.4 ± 0.8	18.9 ± 0.9^#^	24.8 ± 0.9^∗∗^	23.5 ± 1.5^∗^	21 ± 0.9	22 ± 0.5^∗^
	GSH-Px (U/mgprot)	110.5 ± 4.2	98.5 ± 4.3^#^	136.5 ± 8.8^∗∗^	133.4 ± 9.1^∗∗^	114.2 ± 9.8^∗^	114.9 ± 2.1^∗^
	SOD (U/mgprot)	180.4 ± 6.6	135.7 ± 9.6^#^	168.1 ± 11.3^∗^	203 ± 6.8^∗∗^	206 ± 11.6^∗∗^	206 ± 12^∗∗^
	ROS (U/mgprot)	136.6 ± 3.7	151.4 ± 5.1^#^	151 ± 4.7	150.6 ± 8.8	134.3 ± 13.1	121.8 ± 13.5^∗^
	MDA (nmol/mgprot)	8.3 ± 0.2	8.1 ± 0.1	7 ± 0.3^∗^	7.2 ± 0.3^∗^	7.1 ± 0.3^∗^	7.1 ± 0.3^∗^

^#^ *P* < 0.05, ^##^ *P* < 0.01, and ^###^ *P* < 0.001 versus db/m^+^ mice, ^∗^ *P* < 0.05 and ^∗∗^ *P* < 0.01 versus vehicle-treated db/db mice.

## Data Availability

The data used to support the findings of this study are available from the corresponding author upon request.

## Supplementary Materials

Supplementary Materials Table 1s: the primer sequences used in RT-PCR. Table 2s: the detailed parameters of target cytokines decreased or increased over 50% fold among experimental groups, which were measured using the L-series mouse antibody array kit. Among 308 target cytokines related to inflammation, 28 of them were strongly upregulated, and one of them was decreased over 50% fold in vehicle-treated db/db mice compared with db/m^+^ mice. The 50 and 100 mg/kg of SCU for 19 and 24 types of cytokines changed, respectively, compared with vehicle-treated db/db mice. Table 3s: the regulatory effects of SCU and Met on the levels of inflammatory cytokines. The levels of inflammatory cytokines in the serum and/or kidney were measured using enzyme-linked immunosorbent assay kits according to the manufacturer's instructions. Compared with db/m^+^ mice, no significant alterations of several cytokine levels occurred in db/db mice, including MMP-9, TNF-*α*, IL-6, IL-8, ICAM-1, MCP-5, TGF-*β*1/2, and IFN-*β*. Also, SCU treatment for 8 weeks had no influences on those cytokines levels in db/db mice.

## References

[1] Chen F. F., Wang J. T., Zhang L. X. (2017). Oleanolic acid derivative DKS26 exerts antidiabetic and hepatoprotective effects in diabetic mice and promotes glucagon-like peptide-1 secretion and expression in intestinal cells. *British Journal of Pharmacology*.

[2] Zheng T., Yang X., Wu D. (2015). Salidroside ameliorates insulin resistance through activation of a mitochondria-associated AMPK/PI3K/Akt/GSK3*β* pathway. *British Journal of Pharmacology*.

[3] Trasino S. E., Tang X. H., Jessurun J., Gudas L. J. (2016). Retinoic acid receptor *β*2 agonists restore glycaemic control in diabetes and reduce steatosis. *Diabetes, Obesity and Metabolism*.

[4] El Boustany R., Taveau C., Chollet C. (2017). Antagonism of vasopressin V2 receptor improves albuminuria at the early stage of diabetic nephropathy in a mouse model of type 2 diabetes. *Journal of Diabetes and its Complications*.

[5] Cruzat V. F., Keane K. N., Scheinpflug A. L., Cordeiro R., Soares M. J., Newsholme P. (2015). Alanyl-glutamine improves pancreatic *β*-cell function following ex vivo inflammatory challenge. *Journal of Endocrinology*.

[6] Eizirik D. L., Kutlu B., Rasschaert J., Darville M., Cardozo A. K. (2003). Use of microarray analysis to unveil transcription factor and gene networks contributing to *β* cell dysfunction and apoptosis. *Annals of the New York Academy of Sciences*.

[7] Al Hroob A. M., Abukhalil M. H., Alghonmeen R. D., Mahmoud A. M. (2018). Ginger alleviates hyperglycemia-induced oxidative stress, inflammation and apoptosis and protects rats against diabetic nephropathy. *Biomedicine & Pharmacotherapy*.

[8] Jing C., Li X., Zhang J., Wang J. (2013). Responses of the antioxidant system in QGY-7701 cells to the cytotoxicity and apoptosis induced by 1-octyl-3-methylimidazolium chloride. *Journal of Biochemical and Molecular Toxicology*.

[9] Hu M., Ye P., Liao H., Chen M., Yang F. (2016). Metformin protects H9C2 cardiomyocytes from high-glucose and hypoxia/reoxygenation injury via inhibition of reactive oxygen species generation and inflammatory responses: role of AMPK and JNK. *Journal Diabetes Research*.

[10] Pang C., Zheng Z., Shi L. (2016). Caffeic acid prevents acetaminophen-induced liver injury by activating the Keap1-Nrf2 antioxidative defense system. *Free Radical Biology & Medicine*.

[11] Dong W., Jia Y., Liu X. (2017). Sodium butyrate activates NRF2 to ameliorate diabetic nephropathy possibly via inhibition of HDAC. *Journal of Endocrinology*.

[12] Azevedo-Martins A. K., Lortz S., Lenzen S., Curi R., Eizirik D. L., Tiedge M. (2003). Improvement of the mitochondrial antioxidant defense status prevents cytokine-induced nuclear factor-*κ*B activation in insulin-producing cells. *Diabetes*.

[13] Zhou T., Xu X., Du M., Zhao T., Wang J. (2018). A preclinical overview of metformin for the treatment of type 2 diabetes. *Biomedicine & Pharmacotherapy*.

[14] Wang J., Hu W., Li L. (2017). Antidiabetic activities of polysaccharides separated from *Inonotus obliquus* via the modulation of oxidative stress in mice with streptozotocin-induced diabetes. *PLoS One*.

[15] Wang J., Teng L., Liu Y. (2016). Studies on the antidiabetic and antinephritic activities of *Paecilomyces hepiali* water extract in diet-streptozotocin-induced diabetic Sprague Dawley rats. *Journal of Diabetes Research*.

[16] Liu Y., Jing Y. Y., Zeng C. Y. (2018). Scutellarin suppresses NLRP3 inflammasome activation in macrophages and protects mice against bacterial sepsis. *Frontiers in Pharmacology*.

[17] Mo J., Yang R., Li F. (2018). Scutellarin protects against vascular endothelial dysfunction and prevents atherosclerosis via antioxidation. *Phytomedicine*.

[18] Lu K., Han M., Ting H. L., Liu Z., Zhang D. (2013). Scutellarin from *Scutellaria baicalensis* suppresses adipogenesis by upregulating PPAR*α* in 3T3-L1 cells. *Journal of Natural Products*.

[19] Fan H., Ma X., Lin P. (2017). Scutellarin prevents nonalcoholic fatty liver disease (NAFLD) and hyperlipidemia via PI3K/AKT-dependent activation of nuclear factor (erythroid-derived 2)-like 2 (Nrf2) in rats. *Medical Science Monitor*.

[20] Wang J., Tan J., Luo J. (2017). Enhancement of scutellarin oral delivery efficacy by vitamin B12-modified amphiphilic chitosan derivatives to treat type II diabetes induced-retinopathy. *Journal of Nanobiotechnology*.

[21] Long L., Wang J., Lu X. (2015). Protective effects of scutellarin on type II diabetes mellitus-induced testicular damages related to reactive oxygen species/Bcl-2/Bax and reactive oxygen species/microcirculation/staving pathway in diabetic rat. *Journal Diabetes Research*.

[22] Hummel K. P., Dickie M. M., Coleman D. L. (1966). Diabetes, a new mutation in the mouse. *Science*.

[23] Yasuma T., Yano Y., D’Alessandro-Gabazza C. N. (2016). Amelioration of diabetes by protein S. *Diabetes*.

[24] Chow F., Ozols E., Nikolic-Paterson D. J., Atkins R. C., Tesch G. H. (2004). Macrophages in mouse type 2 diabetic nephropathy: correlation with diabetic state and progressive renal injury. *Kidney International*.

[25] Yang S., Liu M., Chen Y. (2018). NaoXinTong capsules inhibit the development of diabetic nephropathy in *db/db* mice. *Scientific Reports*.

[26] Chan L. K. Y., Wang Y., Ng E. K. W., Leung P. S. (2018). Na^+^ /H^+^ exchanger 3 blockade ameliorates type 2 diabetes mellitus via inhibition of sodium-glucose co-transporter 1-mediated glucose absorption in the small intestine. *Diabetes, Obesity and Metabolism*.

[27] Wang J. S., Xie H. T., Zhang M. C. (2017). Characterization of ex vivo expanded oral mucosal epithelium cells on acellular porcine corneal stroma for ocular surface reconstruction. *Journal of Ophthalmology*.

[28] Zheng Y., Zong Z. M., Chen S. L., Chen A. H., Wei X. Y. (2017). Ameliorative effect of *Trametes orientalis* polysaccharide against immunosuppression and oxidative stress in cyclophosphamide-treated mice. *International Journal of Biological Macromolecules*.

[29] Moritoh Y., Takeuchi K., Asakawa T., Kataoka O., Odaka H. (2009). Combining a dipeptidyl peptidase-4 inhibitor, alogliptin, with pioglitazone improves glycaemic control, lipid profiles and beta-cell function in *db/db* mice. *British Journal of Pharmacology*.

[30] Jaakson H., Karis P., Ling K. (2018). Adipose tissue insulin receptor and glucose transporter 4 expression, and blood glucose and insulin responses during glucose tolerance tests in transition Holstein cows with different body condition. *Journal of Dairy Science*.

[31] Li X., Sui Y., Li S., Xie B., Sun Z. (2016). A-type procyanidins from litchi pericarp ameliorate hyperglycaemia by regulating hepatic and muscle glucose metabolism in streptozotocin (STZ)-induced diabetic mice fed with high fat diet. *Journal of Functional Foods*.

[32] Chahil T. J., Ginsberg H. N. (2006). Diabetic dyslipidemia. *Endocrinology and Metabolism Clinics of North America*.

[33] Seibert F. S., Sitz M., Passfall J. (2018). Prognostic value of urinary calprotectin, NGAL and KIM-1 in chronic kidney disease. *Kidney & Blood Pressure Research*.

[34] Huang K. P., Chen C., Hao J., Huang J. Y., Liu P. Q., Huang H. Q. (2015). AGEs-RAGE system down-regulates Sirt1 through the ubiquitin-proteasome pathway to promote FN and TGF-*β*1 expression in male rat glomerular mesangial cells. *Endocrinology*.

[35] Saiman Y., Friedman S. L. (2012). The role of chemokines in acute liver injury. *Frontiers in Physiology*.

[36] Haluzik M., Fulcher G., Pieber T. R., Bardtrum L., Tutkunkardas D., Rodbard H. W. (2018). The co-formulation of insulin degludec and insulin aspart lowers fasting plasma glucose and rates of confirmed and nocturnal hypoglycaemia, independent of baseline glycated haemoglobin levels, disease duration or body mass index: a pooled meta-analysis of phase III studies in patients with type 2 diabetes. *Diabetes Obesity and Metabolism*.

[37] Yang L., Chen C., Li M., Qiu L., Shen J., Bu X. (2018). Effects of anesthesia methods on insulin, blood glucose, immune and postoperative infection of gastric cancer patients complicated with diabetes mellitus. *Minerva Endocrinologica*.

[38] Noguchi T., Inoue H., Tanaka T. (1985). Transcriptional and post-transcriptional regulation of L-type pyruvate kinase in diabetic rat liver by insulin and dietary fructose. *Journal of Biological Chemistry*.

[39] Misawa E., Tanaka M., Nomaguchi K. (2008). Administration of phytosterols isolated from Aloe vera gel reduce visceral fat mass and improve hyperglycemia in Zucker diabetic fatty (ZDF) rats. *Obesity Research & Clinical Practice*.

[40] Yoon J. J., Lee Y. J., Kang D. G., Lee H. S. (2014). Protective role of Oryeongsan against renal inflammation and glomerulosclerosis in db/db mice. *The American Journal of Chinese Medicine*.

[41] Polette M., Nawrocki-Raby B., Gilles C., Clavel C., Birembaut P. (2004). Tumour invasion and matrix metalloproteinases. *Critical Reviews in Oncology/Hematology*.

[42] Novak I., Solini A. (2018). P2X receptor-ion channels in the inflammatory response in adipose tissue and pancreas — potential triggers in onset of type 2 diabetes?. *Current Opinion in Immunology*.

[43] Xu H., Barnes G. T., Yang Q. (2003). Chronic inflammation in fat plays a crucial role in the development of obesity-related insulin resistance. *The Journal of Clinical Investigation*.

[44] Ehses J. A., Perren A., Eppler E. (2007). Increased number of islet-associated macrophages in type 2 diabetes. *Diabetes*.

[45] Liu Y. C., Zou X. B., Chai Y. F., Yao Y. M. (2014). Macrophage polarization in inflammatory diseases. *International Journal of Biological Sciences*.

[46] Sansom D., Borrow J., Solomon E., Trowsdale J. (1991). The human ICAM2 gene maps to 17q23–25. *Genomics*.

[47] Redondo-Castro E., Cunningham C., Miller J. (2017). Interleukin-1 primes human mesenchymal stem cells towards an anti-inflammatory and pro-trophic phenotype in vitro. *Stem Cell Research & Therapy*.

[48] Yang C. M., Luo S. F., Hsieh H. L. (2010). Interleukin-1*β* induces ICAM-1 expression enhancing leukocyte adhesion in human rheumatoid arthritis synovial fibroblasts: involvement of ERK, JNK, AP-1, and NF-*κ*B. *Journal of Cellular Physiology*.

[49] Cardozo A. K., Proost P., Gysemans C., Chen M. C., Mathieu C., Eizirik D. L. (2003). IL-1*β* and IFN-*γ* induce the expression of diverse chemokines and IL-15 in human and rat pancreatic islet cells, and in islets from pre-diabetic NOD mice. *Diabetologia*.

[50] Chen J. H., Huang P. H., Lee C. C., Chen P. Y., Chen H. C. (2013). A bovine whey protein extract can induce the generation of regulatory T cells and shows potential to alleviate asthma symptoms in a murine asthma model. *British Journal of Nutrition*.

[51] Gilbert K. M., Thoman M., Bauche K., Pham T., Weigle W. O. (1997). Transforming growth factor-*β*1 induces antigen-specific unresponsiveness in naive T cells. *Immunological Investigations*.

[52] Zhang R., Xi X., Wang C. (2018). Therapeutic effects of recombinant human interleukin 2 as adjunctive immunotherapy against tuberculosis: a systematic review and meta-analysis. *PLoS One*.

[53] Gorska-Ciebiada M., Saryusz-Wolska M., Borkowska A., Ciebiada M., Loba J. (2016). Adiponectin, leptin and IL-1 *β* in elderly diabetic patients with mild cognitive impairment. *Metabolic Brain Disease*.

[54] Liao P. C., Lai M. H., Hsu K. P. (2018). Identification of *β*-sitosterol as in vitro anti-inflammatory constituent in *Moringa oleifera*. *Journal of Agricultural and Food Chemistry*.

[55] Lee T. W., Kim M. J. (1992). Production of interleukin-2 (IL-2) and expression of IL-2 receptor in patients with IgA nephropathy. *The Korean Journal of Internal Medicine*.

[56] Zavrsnik M., Letonja J., Makuc J., Seruga M., Cilensek I., Petrovic D. (2018). Interleukin-4 gene (IL4) polymorphism rs2243250 is not associated with diabetic nephropathy (DN) in Caucasians with type 2 diabetes mellitus (T2DM). *Bosnian Journal of Basic Medical Sciences*.

[57] Sireesh D., Ganesh M. R., Dhamodharan U. (2017). Role of pterostilbene in attenuating immune mediated devastation of pancreatic beta cells via Nrf2 signaling cascade. *Journal of Nutritional Biochemistry*.

[58] Evans J. L., Goldfine I. D., Maddux B. A., Grodsky G. M. (2003). Are oxidative stress−activated signaling pathways mediators of insulin resistance and *β*-cell dysfunction?. *Diabetes*.

[59] Siti H. N., Kamisah Y., Kamsiah J. (2015). The role of oxidative stress, antioxidants and vascular inflammation in cardiovascular disease (a review). *Vascular Pharmacology*.

[60] Yang H., Jin X., Kei Lam C. W., Yan S. K. (2011). Oxidative stress and diabetes mellitus. *Clinical Chemistry and Laboratory Medicine*.

[61] True A. L., Olive M., Boehm M. (2007). Heme oxygenase-1 deficiency accelerates formation of arterial thrombosis through oxidative damage to the endothelium, which is rescued by inhaled carbon monoxide. *Circulation Research*.

[62] Song Y., Huang L., Yu J. (2016). Effects of blueberry anthocyanins on retinal oxidative stress and inflammation in diabetes through Nrf2/HO-1 signaling. *Journal of Neuroimmunology*.

