# Supplementary material for: Scutellarin Exerts Hypoglycemic and Renal Protective Effects in db/db Mice via the Nrf2/HO-1 Signaling Pathway
Source: Oxid Med Cell Longev. 2019 Feb 10;2019:1354345. doi: 10.1155/2019/1354345 (PMC6387728; doi:10.1155/2019/1354345)
Supplement: Supplementary Materials — Table 1s: the primer sequences used in RT-PCR. Table 2s: the detailed parameters of target cytokines decreased or increased over 50% fold among experimental groups, which were measured using the L-series mouse antibody array kit. Among 308 target cytokines related to inflammation, 28 of them were strongly upregulated, and one of them was decreased over 50% fold in vehicle-treated db/db mice compared with db/m+ mice. The 50 and 100 mg/kg of SCU for 19 and 24 types of cytokines changed, respectively, compared with vehicle-treated db/db mice. Table 3s: the regulatory effects of SCU and Met on the levels of inflammatory cytokines. The levels of inflammatory cytokines in the serum and/or kidney were measured using enzyme-linked immunosorbent assay kits according to the manufacturer's instructions. Compared with db/m+ mice, no significant alterations of several cytokine levels occurred in db/db mice, including MMP-9, TNF-α, IL-6, IL-8, ICAM-1, MCP-5, TGF-β1/2, and IFN-β. Also, SCU treatment for 8 weeks had no influences on those cytokines levels in db/db mice. [file 1354345.f1.pdf]

Supplementary materials

Table.1s: The primer sequences used in RT-PCR

Table.2s: The detail parameters of target cytokines decreased or increased over 50% folds among experimental groups, which were measured using the L-series mouse antibody array kit. Among 308 target cytokines related to inflammation, 28 of them were strongly upregulated, and one of them was decreased over 50% folds in vehicle-treated db/db mice compared with db/m$^+$ mice. The 50 and 100 mg/kg of SCU for 19 and 24 types of cytokines changed, respectively, compared with vehicle-treated db/db mice.

Table.3s: The regulatory effects of SCU and Met on the levels of inflammatory cytokines. The levels of inflammatory cytokines in the serum and/or kidney were measured using enzyme-linked immunosorbent assay kits according to the manufacturer's instructions. Compared with db/m$^+$ mice, no significant alterations of several cytokines levels occurred in db/db mice, including MMP-9, TNF-α, IL-6, IL-8, ICAM-1, MCP-5, TGF-β1/2 and IFN-β. Also, SCU treatment for 8 weeks had no influences on those cytokines levels in db/db mice.

Supplementary table list

Table. 1s The primer sequences used in RT-PCR

Fragment size（bp）	Name	Sequence(5' to 3')
120	RAGE-F	TATTGTCGCTTATACCGCAGTT
	RAGE-R	ACATTTCTTGCCAGGTGGT
126	NGAL-F	GTCAGAGCCCTTGGAAGATG
	NGAL-R	AGAGGAGACAATGTGCAGAAG
75	KIM-1-F	ATCAGATTCAAGTCTTCATTTCAGG
	KIM-1-R	CCTTTACTTCCACATAAGAATCCAC
121	Cd11b-F	CTGAACATCCCATGACCTTCC
	Cd11b-R	CCTGTCTGGTTAACAGCCTTT
78	Cd3-F	AACACGTACTTGTACCTGAAAGCTC
	Cd3-R	GATGATTATGGCTACTGCTGTCA
233	GAPDH-F	GGTGAAGGTCGGTGTGAACG
	GAPDH-R	CTCGCTCCTGGAAGATGGTG

Table.2s The detail parameters of target cytokines decreased or increased over 50% folds among experimental groups.

Coordinate	Target	Fold		
		db/db (vs. db/m+)	db/db+ 50 mg/kg SCU (vs. db/db)	db/db+ 100 mg/kg SCU (vs. db/db)
A 11; A 12	Activin A	57.8%	--	90.2%
A 15; A 16	Activin RIB / ALK-4	--	87.2%	--
B 3; B 4	Axl	164.6%	--	--
B 5; B 6	b FGF	54.5%	--	--
B 13; B 14	beta-Catenin	54.3%	--	--
B 15; B 16	BLC	--	--	119.5%
B 17; B 18	BTC(Betacellulin)	--	--	53.2%
B 19; B 20	Cardiotrophin-1	60.6%	--	--
D 3;D 4	CD40 Ligand / TNFSF5	57.2%	--	--
D 9;D 10	Coagulation Factor III / Tissue Factor	--	--	53.8%
D 13;D 14	CRG-2	235.8%	--	127.5%
D 21;D 22	Csk	51.3%	--	--
E 11; E 12	DAN	--	51.9%	102.6%
E 13; E 14	Decorin	--	57.2%	52.4%
E 15; E 16	DKK-1	--	--	57.9%
F 13; E 14	Eotaxin-2	57.7%	--	--
G 9; G10	FGF R5 beta	--	--	54.3%
G 13; G14	Fit-3 Ligand	97.6%	--	68.1%
G 15; G16	FLRG(Follistain)	--	--	95.1%
G 21; G 22	Frizzled-1	50.2%	--	--
H 1; H 2	G-CSF	105.4%	--	--
H 19; H 20	GITR	-66.2%	176.8%	568.7%
H 27;H 28	Granzyme B	56.8%	-62.5%	--
I 5;I 6	Gremin	--	--	76.0%
I 7;I 8	Growth Hormone R (GHR)	--	--	65.2%
I 11;I 12	HGF	--	67.1%	132.0%
I 17;I 18	ICAM-2/CD102	60.8%	--	--
I 25;I 26	IFN-alpha/beta R2	--	-55.4%	--
J 13;J 14	IGFBP-6	74.0%	--	--
J 17;J 18	IGF-I	55.0%	--	--
J 23;J 24	IL-1 beta	96.0%	--	--
J 27;J 28	IL-1 R6 / IL-1 Rrp2	91.8%	--	--
K 19; K 20	IL-4	325.6%	81.9%	107.4%
K 21; K 22	IL-4 R	86.6%	--	--
L 13; L 14	IL-7 R alpha	--	66.1%	120.0%
L 21; L 22	IL-10 R alpha	63.1%	--	--

L 5; L 6	IL-13 R alpha 2	--	63.6%	72.0%
L 7; L 8	IL-15	--	67.5%	--
L 15; L 16	IL-17BR	89.1%	--	--
M 3;M 4	IL-18 R alpha /IL-1 R5	--	105.4%	67.5%
M 25; M 26	IL-28/IFN-lambda	--	-71.6%	--
M 27; M 28	IL-31	--	55.4%	--
N 1;N 2	IL-31 RA	--	56.0%	--
N 17;N 18	Leptin R	143.2%	--	83.3%
N 19;N 20	LEPTIN(OB)	--	--	52.4%
N 21;N 22	LIF	75.4%	--	--
O 9; O 10	MadCAM-1	--	57.2%	--
P 13;P 14	MCP-5	123.5%	--	--
P 15; P 16	M-CSF	87.1%	--	133.9%
P 25; P 26	MIP-1 alpha	--	-50.2%	--
P 27; P 28	MIP-1 gamma	51.1%	--	--
Q 7; Q 8	MMP-2	--	70.2%	--
R 17;R 18	PIGF-2	--	--	58.9%
R 27;R 28	RANTES	81.9%	--	--
S 13; S 14	Serum Amyloid A1	--	65.0%	62.0%

--: Beyond of the limit (The fold is greater than - 50% and less than 50%).

Table.3s The regulatory effects of SCU and Met on the levels of inflammatory cytokines

		db/m+	db/db	db/db + Met (mg/kg)	db/db + SCU (mg/kg)		
				120	25	50	100
Serum	IFN-β (pg/mL)	319.9±52	344.3±14.1	347.1±27.5	320.7±45.5	333.6±25.8	317.4±14.7
	IL-6 (pg/ml)	67.7±0.9	60.4±1.5	69.9±1.5	65.6±3.2	62.9±1.1	65.6±2
	TNF-α (pg/mL)	32.4±0.4	32.7±0.5	29.3±1.6	32.8±1.5	28.1±2.1	28.1±2.5
	TGF-β1/2 (ng/mL)	118.3±1.2	115±1.1	113.6±2.7	117.5±3.5	119.8±1.7	118.4±5.6
	MMP-9 (ng/mL)	52±0.8	49.8±0.5	49.1±0.7	50±1.5	51.6±1	52.5±1.6
Kidney	IL-6 (pg/mgprot)	49.1±1.1	49.1±1.2	44.4±0.9	35.6±1.3	32.8±0.6	33.8±1.8
	IL-8 (pg/mgprot)	31.5±1.1	31.1±1.7	35.7±1.3	31.7±3.6	30±3.1	28.5±0.5
	TNF-α (pg/ mgprot)	13.7±0.3	15±0.4	17.3±2.2	14.2±0.4	13.5±0.3	13.4±0.6
	ICAM-1 (ng/ mgprot)	103.8±4.7	102.8±4.1	114.9±5.3	115.6±9.7	97.1±2.9	101.1±2.9
	ICAM-2 (nmol/ mgprot)	21.7±0.5	20.5±0.1	22.2±1.1	16.4±0.6	17±0.5	16.5±0.5
	MCP-5 (pg/mgprot)	133.5±8	127.4±5.8	148.2±3	127.4±11.1	154.1±2.5	133.5±3.2
	MMP-9 (ng/ mgprot)	24.8±0.7	22.5±0.2	23.7±0.5	19.8±0.9	20.1±0.6	20.2±0.8
